# Risk Factors for Suicide in Patients With Head and Neck Squamous Cell Carcinoma

**DOI:** 10.7759/cureus.100638

**Published:** 2026-01-02

**Authors:** Tatsuji Nishiguchi, Ichiro Tojima, Kai Yamazaki, Kento Kawakita, Yoshihito Kubo, Tomoyuki Sudo, Koji Matsumoto, Shigehiro Owaki, Yukinori Takenaka

**Affiliations:** 1 Otolaryngology - Head and Neck Surgery, Shiga University of Medical Science, Otsu, JPN

**Keywords:** cancer mortality, cancer of the head and neck, hypopharynx cancer, squamous cell carcinoma, suicide

## Abstract

Background

Head and neck squamous cell carcinoma (HNSCC) is associated with a high risk of suicide, partly because of the functional morbidity caused by the disease and its treatment. This study aimed to identify risk factors for suicide among patients with HNSCC, focusing on the subsites of the primary tumor.

Methodology

Using the Surveillance, Epidemiology, and End Results database, we obtained data from 212,675 cases diagnosed as HNSCC. Causes of death were categorized as suicide, cancer, or non-cancer-related. Cumulative incidence functions were used to estimate cause-specific mortality, and Fine-Gray competing risks models were applied to assess prognostic factors. Weighted linear regression was performed to examine associations between site-specific suicide risk and cancer/non-cancer mortality across subsites.

Results

During follow-up, 708 patients died by suicide. The five- and ten-year cumulative incidence of suicide was 0.3% and 0.4%, respectively. Suicide incidence varied by primary site, with hypopharyngeal cancer demonstrating the highest rate (123.6 per 100,000 person-years) and nasopharyngeal cancer the lowest (25.3 per 100,000 person-years). Multivariate analysis revealed hypopharyngeal primary tumor as an independent risk factor for suicide (subdistribution hazard ratio = 2.30, 95% confidence interval = 1.01-5.23, p = 0.046), along with male sex and earlier year of diagnosis. Although hypopharyngeal cancer also exhibited high cancer mortality, weighted regression demonstrated no significant association between site-specific suicide and cancer mortality risks (p = 0.235). Similarly, suicide risk did not correlate with non-cancer mortality (p = 0.112).

Conclusions

Hypopharyngeal cancer was independently associated with a high risk of suicide. However, suicide risk did not parallel cancer prognosis across primary sites. Further studies are required to determine specific risk factors for suicide and develop preventive measures.

## Introduction

Head and neck cancer is the seventh most common cancer worldwide [1]. Various histological types of cancer occur in the head and neck region, among which squamous cell carcinoma (SCC) is the most common [1]. Head and neck squamous cell carcinoma (HNSCC) accounts for 90% of head and neck cancers [1], and 97% of the HNSCC cases arise in the upper aerodigestive tract [2], which causes various symptoms that deteriorate the quality of life of patients. Specifically, oral cavity cancers frequently impair mastication, speech, and swallowing; nasopharyngeal cancers are often associated with hearing loss and cranial nerve dysfunction; oropharyngeal cancers may affect swallowing and voice; hypopharyngeal and laryngeal cancers commonly cause dysphonia and dysphagia; and paranasal cancer may result in facial disfigurement [1]. These cancer-related symptoms, in conjunction with adverse events from treatment and financial burden, often cause social disability and, in extreme cases, result in suicide [3,4].

Previous studies have shown an increased incidence of suicide among patients with cancer or cancer survivors compared to the general population [5,6]. Among cancers of various organs, head and neck cancer is a high-risk factor for suicide, with an approximately 2.4-fold higher risk compared with non-head and neck cancers [3]. Other than head and neck origin, female sex, rural residence, and marital status have been reported as risk factors for suicide in patients with cancer [3-8]. In addition, previous research has shown a higher suicide rate among patients with cancers with higher mortality rates [5,6]. However, important gaps remain in the previous studies. Most prior studies have evaluated suicide risk using standardized mortality ratios compared with the general population [3-8], which limits the ability to compare suicide risk across different head and neck cancer subsites while adjusting for competing causes of death and other confounders. Moreover, suicide is a rare outcome, and even multi-institutional cohort studies [9] would be underpowered to draw robust conclusions regarding subsite-specific risk. As a result, it remains unclear whether suicide risk simply parallels cancer mortality across head and neck subsites or whether certain subsites confer an excess risk related to site-specific functional impairment. On the other hand, voice loss is a large burden for patients undergoing laryngectomy [10]. Based on these, we hypothesized that the suicide rate would be elevated in patients with high mortality head and neck subsites and that patients with laryngeal and hypopharyngeal cancers, who are candidates for laryngectomy, might have higher suicide rates than expected from their mortality rates. Due to the small incidence of suicide, even multi-institutional studies [5,6] could not offer a scientifically sound conclusion. To address this limitation, we conducted a population-based study. The objective of this study was to evaluate subsite-specific suicide risk among patients with HNSCC using a large population-based registry, while accounting for competing risks of cancer-related and non-cancer-related mortality.

## Materials and methods

This study was a retrospective, population-based secondary analysis of data obtained from the Surveillance, Epidemiology, and End Results (SEER) program. The SEER database comprises de-identified, publicly available cancer registry data collected from population-based registries in the United States. All patients diagnosed with HNSCC between January 2000 and December 2022 were eligible for inclusion. Because the SEER data are fully anonymized and publicly available, institutional review board approval and informed consent were not required.

Data retrieval

The SEER database is a population-based registry in which all cancer cases diagnosed within the covered geographic areas are systematically recorded. Therefore, no sampling technique was applied; all eligible cases that met the predefined inclusion criteria were included in the analysis.

Individual patient data were retrieved from the database: Incidence - SEER Research Plus Data, 17 Registries, November 2024 Sub (2000-2022) [11], which collects cancer cases from 17 U.S. regions and covers 26.5% of the U.S. population, using SEER Stat software, version 9.0.42. (National Cancer Institute, Bethesda, MD, USA). We defined HNSCC as cases meeting the following criteria: (1) primary site in the head and neck; ICD-O-3 site codes C01.9-base of the tongue, NOS,C02.0-dorsal surface of the tongue, NOS,C02.1-border of the tongue, C02.2-ventral surface of the tongue, NOS,C02.3-anterior two-thirds of the tongue, NOS,C02.4-lingual tonsil, C02.8-overlapping lesion of the tongue, C02.9-tongue, NOS,C03.0-upper gum, C03.1-lower gum, C03.9-gum, NOS,C04.0-anterior floor of the mouth, C04.1-lateral floor of the mouth, C04.8-overlapping lesion of the floor of the mouth, C04.9-floor of the mouth, NOS,C05.0-hard palate, C05.1-soft palate, NOS,C05.2-uvula, C05.8-overlapping lesion of the palate, C05.9-palate, NOS,C06.0-cheek mucosa, C06.1-vestibule of the mouth, C06.2-retromolar area, C06.8-overlapping lesion of other and unspecified mouth, C06.9-mouth, NOS,C07.9-parotid gland, C08.0-submandibular gland, C08.1-sublingual gland, C08.8-overlapping lesion of major salivary glands, C08.9-major salivary gland, NOS,C09.0-tonsillar fossa, C09.1-tonsillar pillar, C09.8-overlapping lesion of the tonsil, C09.9-tonsil, NOS,C10.0-vallecula, C10.1-anterior surface of the epiglottis, C10.2-lateral wall of the oropharynx, C10.3-posterior wall of the oropharynx, C10.4-branchial cleft, C10.8-overlapping lesion of the oropharynx, C10.9-oropharynx, NOS,C11.0-superior wall of the nasopharynx, C11.1-posterior wall of the nasopharynx, C11.2-lateral wall of the nasopharynx, C11.3-anterior wall of the nasopharynx, C11.8-overlapping lesion of the nasopharynx, C11.9-nasopharynx, NOS,C12.9-pyriform sinus, C13.0-postcricoid region, C13.1-aryepiglottic fold, hypopharyngeal, C13.2-posterior wall of the hypopharynx, C13.8-overlapping lesion of the hypopharynx, C13.9-hypopharynx, NOS,C30.0-nasal cavity, C31.0-maxillary sinus, C31.1-ethmoid sinus, C31.2-frontal sinus, C31.3-sphenoid sinus, C31.8-overlapping lesion of accessory sinuses, C31.9-accessory sinus, NOS,C32.0-glottis, C32.1-supraglottis, C32.2-subglottis, C32.3-laryngeal cartilage, C32.8-overlapping lesion of the larynx, C32.9-larynx, NOS. (2) Histologically confirmed SCC; ICD-O-3 histology codes 8070/3: squamous cell carcinoma, NOS,8071/3: squamous cell carcinoma, keratinizing, NOS,8072/3: squamous cell carcinoma, large cell, nonkeratinizing, NOS,8073/3: squamous cell carcinoma, small cell, nonkeratinizing, 8074/3: squamous cell carcinoma, spindle cell, 8075/3: squamous cell carcinoma, adenoid, 8076/3: squamous cell carcinoma, micro-invasive, 8077/3: squamous cell carcinoma, grade III, 8078/3: squamous cell carcinoma with horn formation, 8083/3: basaloid squamous cell carcinoma, 8084/3: squamous cell carcinoma, clear cell type, 8085/3: squamous cell carcinoma, HPV-positive, and 8086/3: squamous cell carcinoma, HPV-negative. The exclusion criteria were as follows: (1) unknown tumor extension and (2) unknown causes of death. Patients with a history of prior malignancy were included in the analysis, and HNSCC was analyzed as the index cancer for defining the primary site.

Data on histology, age at diagnosis, year of diagnosis, sex, race, primary sites, SEER summary stage, follow-up period, and cause of death were retrieved. The SEER summary stage is a basic staging classification that categorizes tumor extent as localized, regional, or distant. Cause of death was classified and recorded according to ICD-10 codes in the SEER database. Suicide was defined as U03 (suicide terrorism), X60-X84 (intentional self-harm), and Y87.0 (sequelae of intentional self-harm).

Statistical analysis

The causes of death were classified as suicide, cancer, and other causes. Suicide incidence per 100,000 person-years was calculated by dividing the number of suicide deaths by the total person-years at risk and multiplying by 100,000. The cumulative incidence rates for suicide, cancer mortality, and other cause mortality were calculated using non-parametric cumulative incidence functions. Multivariate analysis of cumulative incidence was performed using the Fine and Gray subdistribution models. Statistical significance was set at p-values <0.05. As an exploratory analysis to examine associations among suicide, cancer mortality, and other-cause mortality, we employed weighted linear regression using log-transformed subdistribution hazard ratios (sHRs) or mortality rates for each cancer site. Each observation was weighted by the inverse of the variance estimated from the 95% confidence interval (CI) of the outcome. All statistical analyses were performed using EZR [12] (Jichi Medical University, Saitama, Japan), a graphical interface of the R software (R Foundation for Statistical Computing, Vienna, Austria).

## Results

Patient characteristics

The SEER database contained 236,179 cases of HNSCC. After applying the exclusion criteria, 212,675 patients were included in this study. Table 1 shows the patient characteristics of this cohort. The male-to-female ratio was 3.02. The most common site was the oropharynx, followed by the larynx and oral cavity. The tumor extent was local in 35.1%, regional in 48.2%, and distant in 16.7% of patients. One-fourth of the patients had a previous history of cancer. The median follow-up period for surviving patients was 5.4 years (65 months).

**Table 1:** Patient characteristics. SD: standard deviation

N = 212,675		Number of patients (%)
Age	(Mean ± SD)	64.0 ± 12.0
Sex	Male	159,619 (75.1)
	Female	53,056 (24.9)
Race (%)	White	177,419 (83.4)
	Black	20,866 (9.8)
	Asian or Pacific Islander	11,946 (5.6)
	Other	2,444 (1.1)
Site (%)	Oral	55,210 (26.0)
	Nasopharynx	5,988 (2.8)
	Oropharynx	76,795 (36.1)
	Hypopharynx	9,008 (4.2)
	Larynx	55,914 (26.3)
	Nose and sinus	5,077 (2.4)
	Major salivary glands	4,683 (2.2)
Tumor extent	Local	74,752 (35.1)
	Regional	102,466 (48.2)
	Distant	35,457 (16.7)
Previous history of cancer	No	170,312 (80.1)
	Yes	42,363 (19.9)
Year of diagnosis	2000–2004	27,279 (12.8)
	2005–2009	43,497 (20.5)
	2010–2014	50,255 (23.6)
	2015–2019	56,132 (26.4)
	2020–2022	35,512 (16.7)
Median follow-up period among survivors, years (months)		5.4 (65)

Incidence of mortality according to causes

**Figure 1:**
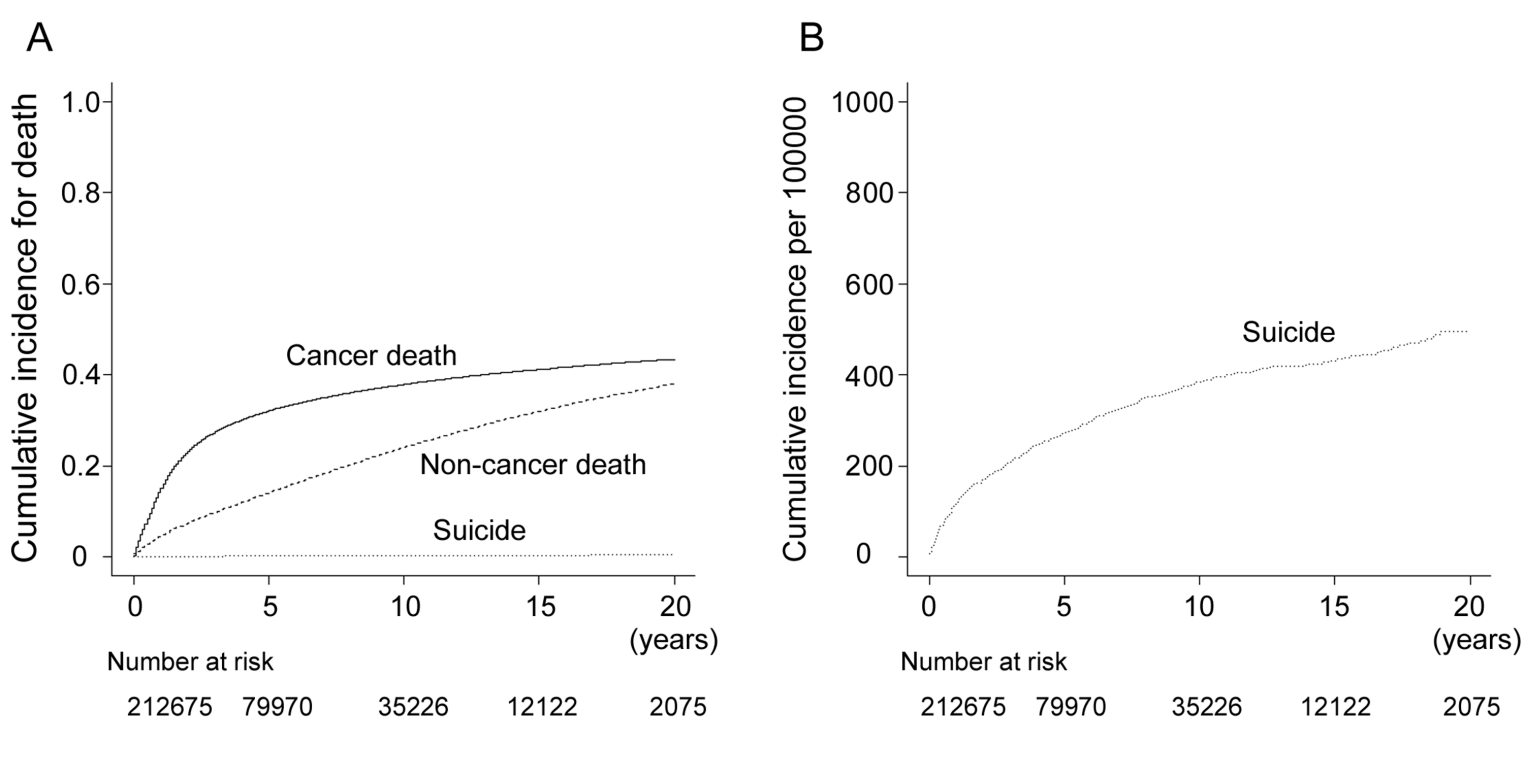
Cumulative incidence for death. (A) Cumulative incidence for cancer mortality, non-cancer mortality, and suicide. (B) Cumulative incidence for suicide per 100,000.

During the follow-up period, 72,080 patients died of cancer, 708 died by suicide, and 45,625 died of other causes. Figure 1A and Figure 1B show the cumulative incidence of mortality. The five- and ten-year cumulative incidences were 32.2% and 37.9% for cancer mortality, 0.3% and 0.4% for suicide, and 14.2% and 24.1% for other-cause mortality, respectively (Table 2).

**Table 2:** Cumulative incidence by cause of death. CI: confidence interval

	Suicide		Cancer mortality		Non-cancer mortality	
	(%)	95% CI	(%)	95% CI	(%)	95% CI
5-year cumulative incidence	0.3	0.3–0.4	32.2	31.9–32.4	14.2	14.0–14.4
10-year cumulative incidence	0.4	0.2–0.3	37.9	37.7–38.2	24.1	23.9–24.3

Suicide according to primary sites

**Figure 2:**
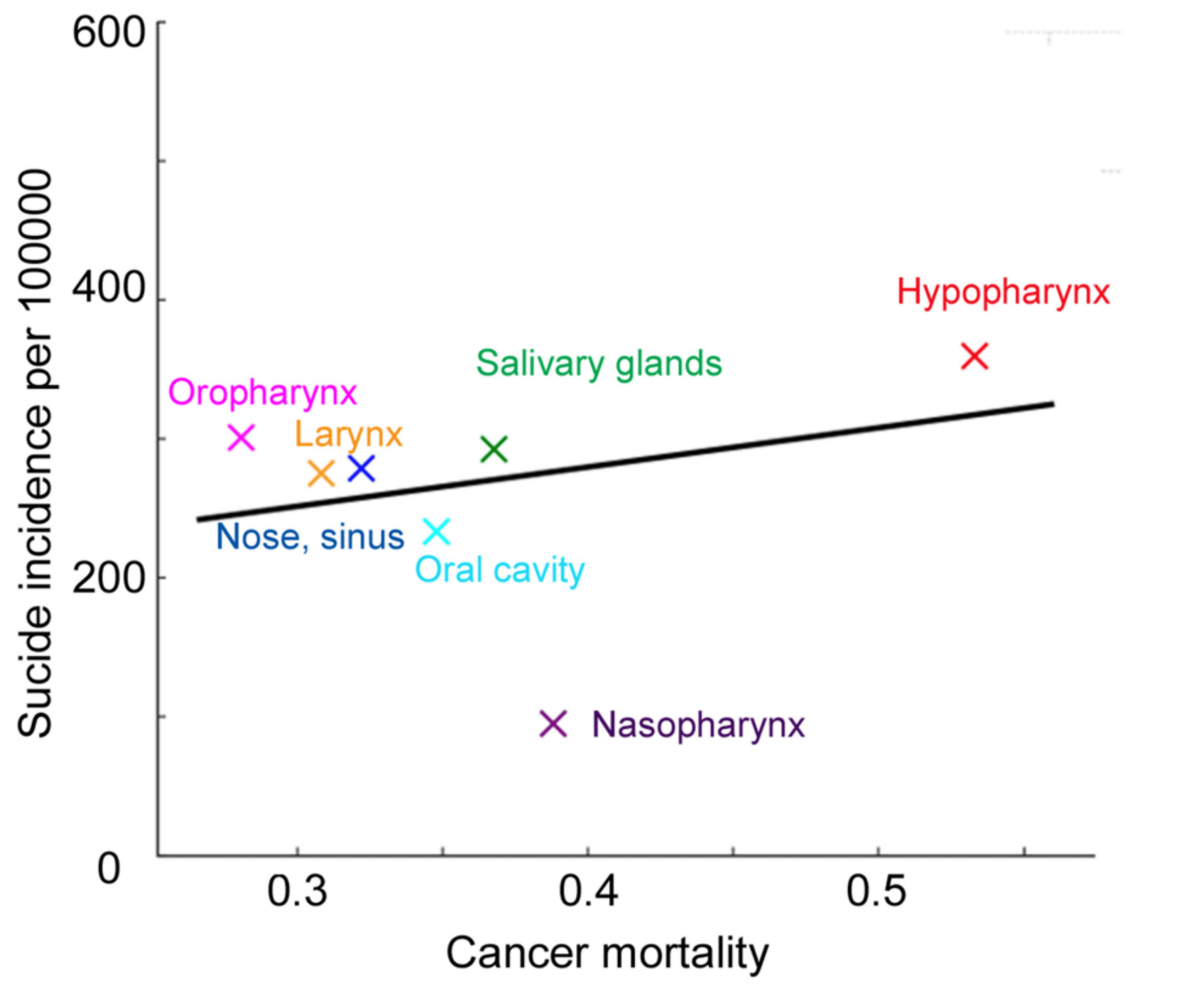
Relationship between suicide and cancer mortality. Cumulative suicide incidence per 100,000 and cumulative incidence for cancer mortality at five years in each head and neck site is plotted. A regression line is shown in black.

Suicide per 100,000 person-years was 123.6 for hypopharynx, 72.6 for salivary gland, 70.9 for oropharynx, 67.7 for larynx, 60.8 for oral cavity, 60.3 for nasal cavity and sinuses, and 25.3 for nasopharynx. Figure 2 shows the association between the five-year suicide and cancer mortality rates. Hypopharyngeal cancer had the highest rates of both suicide and cancer mortality. Overall, higher cancer mortality tended to coincide with higher suicide rates, although nasopharyngeal cancer appeared to be an outlier (correlation coefficients: 0.204 including nasopharynx and 0.704 excluding nasopharynx). Weighted linear regression analyses showed no significant association between the suicide rate and cancer mortality (p = 0.730, including all primary sites; p = 0.503, excluding the nasopharynx).

Factors associated with suicide, cancer, and other-cause mortality

**Figure 3:**
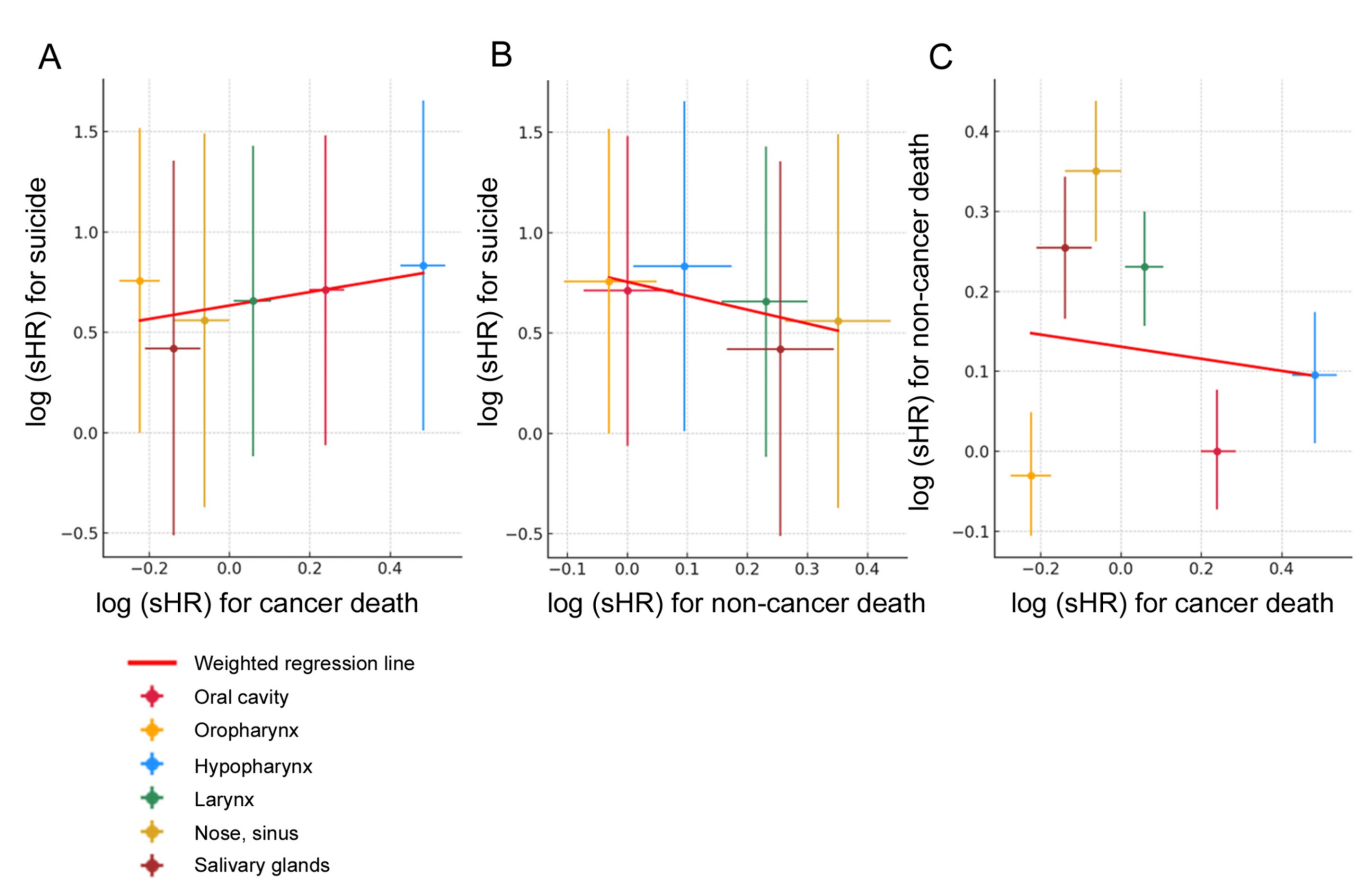
Relationship between causes of death. (A) Suicide and cancer mortality. (B) Suicide and non-cancer mortality. (C) Non-cancer mortality and cancer mortality. Logarithmized subdistribution hazard ratio for each head and neck site are plotted and regression lines are shown. sHR: subdistribution hazard ratio

Table 3 presents the results of the Fine and Gray subdistribution hazard analyses. Hypopharyngeal primary was an independent prognostic factor for suicide (subdistribution hazard ratio 2.30 (95% CI = 1.01-5.23, p = 0.046). Although oropharyngeal primary showed a tendency toward lower suicide, there was no significant association (p = 0.051). Additionally, sex and year of diagnosis were independently associated with the subdistribution hazard of suicide. Specifically, female sex was associated with a lower risk of suicide, and the risk of suicide decreased with more recent year of diagnosis. In contrast, several primary sites were favorable or adverse prognostic factors for cancer and non-cancer mortality. Next, we investigated the association between suicide, cancer, and non-cancer mortality (Figure 3). The weighted regression between suicide and cancer mortality yielded a slope of 0.61 (95% CI = -0.50 to 1.71, p = 0.235) (Figure 3A). The weighted regression between suicide and non-cancer mortality yielded a slope of -0.69 (95% CI = -1.64 to 0.25, p = 0.112) (Figure 3B). No association was found between cancer-related and non-cancer-related mortality (p = 0.797) (Figure 3C). Thus, there was no significant association between suicide and cancer-related mortality or between suicide and non-cancer-related mortality.

**Table 3:** Subditribution hazard ratio for mortality. CI: confidence interval; sHR: subdistribution hazard ratio

		Suicide			Cancer mortality			Non-cancer mortality		
		sHR	95% CI (upper limit–lower limit)	P-value	sHR	95% CI (upper limit–lower limit)	P-value	sHR	95% CI (upper limit–lower limit)	P-value
Age		0.9993	0.99–1.01	0.850	1.02	1.02–1.02	<0.001	1.04	1.04–1.04	<0.001
Sex	Male	Reference			Reference			Reference		
	Female	0.1997	0.15–0.27	<0.001	1.04	1.02–1.05	<0.001	0.91	0.89–0.93	<0.001
Race	White	1.537	0.64–3.71	0.340	1.11	1.03–1.20	0.009	1.12	1.01–1.25	0.032
	Black	0.4486	0.17–1.18	0.110	1.57	1.45–1.70	<0.001	1.25	1.12–1.39	<0.001
	Asian or Pacific Islander	0.6399	0.23–1.76	0.390	1.12	1.02–1.22	0.012	0.77	0.68–0.86	<0.001
	Other	Reference			Reference			Reference		
Tumor extent	Local	Reference			Reference			Reference		
	Regional	0.9326	0.77–1.14	0.490	2.15	2.11–2.19	<0.001	0.86	0.83–0.88	<0.001
	Distant	0.8863	0.78–1.00	0.055	3.93	3.84–4.01	<0.001	0.73	0.71–0.76	<0.001
Primary site	Oral cavity	2.04	0.94–4.4	0.070	1.27	1.22–1.33	<0.001	1	0.93–1.08	0.99
	Nasopharynx	Reference			Reference			Reference		
	Oropharynx	2.13	1.0–4.56	0.051	0.8	0.76–0.84	<0.001	0.97	0.9–1.05	0.44
	Hypopharynx	2.30	1.01–5.23	0.046	1.62	1.53–1.71	<0.001	1.1	1.01–1.19	0.034
	Larynx	1.93	0.89–4.18	0.096	1.06	1.01–1.11	0.018	1.26	1.17–1.35	<0.001
	Nose and sinus	1.75	0.69–4.44	0.240	0.94	0.87–1.0	0.054	1.42	1.3–1.55	<0.001
	Major salivary glands	1.52	0.6–3.88	0.380	0.87	0.81–0.93	<0.001	1.29	1.18–1.41	<0.001
Previous history of cancer	No	Reference			Reference			Reference		
	Yes	1.054	0.86–1.28	0.6	0.92	0.90–0.93	<0.001	0.74	0.72–0.75	<0.001
Year of diagnosis	2000–2004	Reference			Reference			Reference		
	2005–2009	1.16	0.92–1.46	0.210	0.86	0.84–0.88	<0.001	0.91	0.89–0.93	<0.001
	2010–2014	1.015	0.90–1.14	0.810	0.76	0.74–0.77	<0.001	0.76	0.74–0.78	<0.001
	2015–2019	0.9318	0.86–1.01	0.095	0.65	0.63–0.66	<0.001	0.61	0.60–0.63	<0.001
	2020–2022	0.8677	0.79–0.95	0.003	0.62	0.60–0.64	<0.001	0.46	0.44–0.48	<0.001

## Discussion

Owing to advances in treatment, the prognosis of cancer has improved. According to the 2023 Cancer Statistics Report, the cancer mortality rate declined by 33% since 1991 [13]. Consequently, preventable deaths other than cancer have become an important issue for cancer survivors. Globally, 9.4 per 100,000 people die by suicide [14]. Among patients diagnosed with cancer, the suicide rate is higher than that in the general population, and the standardized mortality ratio (SMR) is approximately 1.2 [5,6]. Additionally, cancer types with poor prognoses have been shown to be risk factors for suicide [6]. This prompted us to examine whether a poorer prognosis is associated with higher suicide rates across different head and neck cancer sites. Furthermore, different head and neck sites confer different life-changing symptoms and treatment-related comorbidities. For example, oral cavity cancers frequently result in impaired mastication, speech, and swallowing due to surgical resection and reconstruction [1,15]. Oropharyngeal cancers are commonly associated with dysphagia and long-term feeding tube dependence following chemoradiation, even in patients with favorable oncologic outcomes [1,16]. Hypopharyngeal and laryngeal cancers often cause severe dysphonia or permanent voice loss, particularly in patients undergoing total laryngectomy, which has been shown to substantially impair quality of life and social functioning [10]. In addition, cancers of the nasal cavity, paranasal sinuses, and major salivary glands may lead to facial disfigurement, sensory deficits, or xerostomia, further contributing to long-term functional and psychosocial burden [1,15]. Therefore, we examined whether specific head and neck sites were outliers. We found that the suicide incidence was the lowest in nasopharyngeal cancer. Furthermore, we demonstrated that after adjusting for confounding factors, hypopharyngeal cancer was associated with a high risk of suicide and cancer mortality. However, no significant association was found between suicide and cancer mortality across the head and neck sites.

Several studies have investigated suicide risk across head and neck sites, and systematic reviews and meta-analyses have been conducted [3,4]. Nassar et al reported the high risk of suicide among oropharyngeal and laryngeal cancers [3]. In contrast, Sharma et al. reported the highest suicide risk among patients with oral cancer, followed by those with nasopharyngeal, laryngeal, and hypopharyngeal cancers [4]. Our results contradict those of these meta-analyses. One reason for this is the methods used to evaluate suicide risk. Most previous studies on suicide used the SMR to evaluate suicide risk [3-7]. SMR is effective for comparing suicide risk between patients with cancer and the general population because it adjusts for age, sex, and race [17]. However, when comparing suicide risk across different cancer types, multivariate analyses, which enable adjustment for many factors, would be more appropriate. In our study, we employed a Fine and Gray model for multivariate analyses because it accounts for competing mortality. In addition, differences in cohort size, population demographics, and regional clinical practices, including treatment approaches, may also contribute to the observed discrepancies. Many of the previous meta-analyses included relatively small cohort studies [3,4]. Given that suicide is a rare event, very large cohort studies, particularly population-based studies, are better suited to accurately estimate suicide risk. Another reason is that these meta-analyses included relatively small cohort studies. Suicide is a rare event; therefore, very large cohort studies, i.e., population-based studies, could accurately estimate the exact risk. The other reason is that we limited our analysis to patients with SCC to reduce heterogeneity. Even when cancer arises from the same organ, treatment modalities and prognosis differ among the different histologic types of cancer, which results in varying suicide rates. Of note, nasopharyngeal cancer appeared as an outlier when the association between suicide rate and cancer mortality was examined (Figure 2). Nasopharyngeal cancer has a unique background in that it is likely to arise in the East and Southeast Asian ethnicities [18]. Moreover, nasopharyngeal cancer is typically treated non-surgically in contrast to other HNSCC, for which surgery is a major option. Further, nasopharyngeal carcinoma is generally more responsive to chemotherapy and radiation therapy compared with other HNSCC. These characteristics may partly explain why nasopharyngeal cancer appeared as an outlier in our analysis.

The prevention of suicide in patients with cancer has become more important as mortality from cancer and other causes decreases (Table 2). Advocated strategies for suicide prevention include screening for depression and suicidality, social support, psychological intervention, and prophylactic antidepressants [14]. Additionally, reducing treatment-related functional loss is important. While HNSCC itself can cause dysphagia, dysphonia, and other functional impairments, survivors often experience additional treatment-related functional loss. Surgical treatment may result in persistent dysphagia and dysphonia, radiation therapy is commonly associated with xerostomia and swallowing dysfunction, and chemotherapy can lead to hearing loss and peripheral neuropathy [1]. To reduce these treatment-related toxicities, risk-based de-intensification treatment strategies have been investigated for HNSCC, particularly in human papillomavirus-associated oropharyngeal carcinoma [16,19]. De-intensification strategies are broadly classified into two approaches: reduction of radiation dose and reduction or modification of chemotherapeutic drugs. When these risk-adapted strategies are introduced in clinical practice, the number of patients with lasting toxicities will decrease, which may result in a reduction in suicide rates. Furthermore, reducing late toxicities through de-intensified treatment might also decrease non-cancer mortality, which accounts for a substantial proportion of deaths in patients with HNSCC [20].

Our study has several limitations. First, the SEER database does not include some potentially confounding data, including comorbidities, social history, and socioeconomic status. Comorbidities, especially psychiatric disorders, affect suicidality [4]. Alcohol abuse is also associated with a higher suicide risk [4]. Furthermore, education, employment status, poverty, and living areas are associated with suicide risk among patients with cancer [21]. Second, oncologic outcomes other than the causes of death were lacking. Persistent disease or recurrence after treatment can be more depressing than the initial cancer diagnosis [22]. However, due to a lack of data, we could not examine the association between recurrence and suicide. Third, functional loss after cancer treatment was not assessed. Functional loss after HNSCC treatment, including loss of voice, feeding tube dependence, and facial disfigurement, may be sufficient for some patients to develop suicidal ideation [15]. Assessing the association between functional status and suicide is vital for considering future cancer treatment strategies. Fourth, cause-of-death coding in registry data is susceptible to misclassification. In particular, deaths due to injury or drug overdose may be coded as accidental or of undetermined intent rather than intentional self-harm. Such misclassification would be expected to result in underestimation of the suicide rate and biased effect estimates toward the null. Lastly, this study was based on the SEER database, which is a population-based registry in the United States. Therefore, the results presented in this study may not be generalizable to populations in other countries with different ethnicities, cultures, and healthcare systems. Additionally, although the SEER 17 covers about 30% of the U.S. population, the remaining 70% is not represented, which may introduce selection bias. Accordingly, caution is warranted when extrapolating our findings to other settings.

Despite these limitations, this study has several important strengths. First, we used a large population-based registry with long-term follow-up, which allowed robust estimation of suicide risk in a rare outcome that cannot be adequately studied in smaller cohorts. Second, by applying competing-risk methods, we appropriately accounted for cancer-related and non-cancer-related mortality, providing a more accurate assessment of suicide risk. Third, our subsite-specific analysis highlights heterogeneity in suicide risk across head and neck cancer sites, offering insights beyond prior studies that primarily relied on comparisons with the general population.

## Conclusions

This study identified hypopharyngeal primary cancer as a significant risk factor for suicide. Notably, and contrary to some previous reports, we found that a poorer oncological prognosis does not necessarily correlate with a higher risk of suicide. This discrepancy likely stems from the fact that each head and neck primary site imposes distinct functional losses and treatment sequelae; likewise, different treatment modalities carry unique complications and toxicities. Consequently, future research must account for these functional impairments and their impact on quality of life. To truly improve patient outcomes, it is essential to develop integrated treatment strategies that target the reduction of suicide risk alongside cancer-specific and non-cancer mortality.
